# Co-Deposited Proteins in Alzheimer’s Disease as a Potential Treasure Trove for Drug Repurposing

**DOI:** 10.3390/molecules30081736

**Published:** 2025-04-13

**Authors:** Avgi E. Apostolakou, Dimitra E. Douska, Zoi I. Litou, Ioannis P. Trougakos, Vassiliki A. Iconomidou

**Affiliations:** Section of Cell Biology and Biophysics, Department of Biology, School of Science, National and Kapodistrian University of Athens, Panepistimiopolis, 157 01 Athens, Greece; avapo@biol.uoa.gr (A.E.A.); zlitou@biol.uoa.gr (Z.I.L.); itrougakos@biol.uoa.gr (I.P.T.)

**Keywords:** Alzheimer’s disease, amyloids, drug repurposing, networks, protein–protein interactions

## Abstract

Alzheimer’s disease (AD) affects an increasing number of people as the human population ages. The main pathological feature of AD, amyloid plaques, consists of the key protein amyloid-β and other co-deposited proteins. These co-deposited proteins and their protein interactors could hold some additional functional insights into AD pathophysiology. For this work, proteins found on amyloid plaques were collected from the AmyCo database. A protein–protein and protein–drug interaction network was constructed with data from the IntAct and DrugBank databases, respectively. In total, there were 12 proteins co-deposited on amyloid plaques that reportedly interact with 513 other proteins and are targets of 72 drugs. These drugs were shown to be almost entirely distinct from the panel of drugs currently approved by the FDA for AD and their corresponding protein targets. In conclusion, this work demonstrates the potential for drug repurposing of drugs that target proteins found in amyloid plaques.

## 1. Introduction

As larger portions of the human population are aging, late-onset diseases begin to present an ever more emergent challenge. Alzheimer’s Disease (AD), the most common form of dementia, is predicted to potentially affect over 100 million people globally by 2050 [1]. Being a complex neurodegenerative disease, AD is characterized by two pathological hallmarks, namely amyloid plaques and neurofibrillary tangles [2]. The cause of AD is considered to be multifactorial, with both environmental and genetic factors at play [3]. A key factor in AD is amyloid-β, whose critical role is generally well accepted [4]; thus, attempts to find effective treatments have focused mostly on amyloid-β, but have only recently begun to show limited success.

Organized extracellular protein aggregates, or amyloids, have been linked to a number of diseases, often as a cause [5]. Amyloid deposits are composed of a key protein, that in the case of amyloid plaques is amyloid-β, and other components referred to as “amyloid signature proteins” [6] or “co-deposited proteins” [7]. The importance of co-deposited proteins is unclear and remains under investigation. In AD, such proteins have been implicated in the regulation of amyloid formation, such as the molecular chaperone clusterin [8,9] and the product of the genetic risk factor apolipoprotein E (APOE) [10]; however the precise mechanisms underlying the role of most proteins is still unclear. Exploring these proteins and their potential as modulators has both mechanistic and pharmacological interest.

Studying complex systems like the mechanisms underlying amyloidogenesis and disease requires among others the use of bioinformatic approaches. Specifically, networks have been successfully employed many times, with uses including the identification of disease genes [11] as well as the uncovering of common elements in amyloid formation [12]. A relevant application of networks is to assist in drug discovery, a process that is at once long, risky, and costly [13]. To mitigate these issues, a strategy called drug repurposing or repositioning can be used, where existing drugs are used beyond their original scope for development [14]. A number of studies have utilized networks to explore drug repurposing in AD or similar approaches, each with different methodology and reporting results [15,16,17,18,19,20].

The aim of this work was to closely examine the therapeutic potential for AD of the previously mostly overlooked proteins found on amyloid plaques, i.e., amyloid-β and co-deposited proteins. A network of both protein–protein and protein–drug interactions was constructed using these proteins as a seed set. Each drug targeting one of these proteins was reviewed for links to AD. In addition, a network of FDA-approved drugs for AD and their targets was created and contrasted against. Finally, a suggestion is made for further study of potential protein targets and drugs with a potential for repurposing in AD.

## 2. Results

### 2.1. FDA Approved Drugs and Their Protein Targets

**Figure 1:**
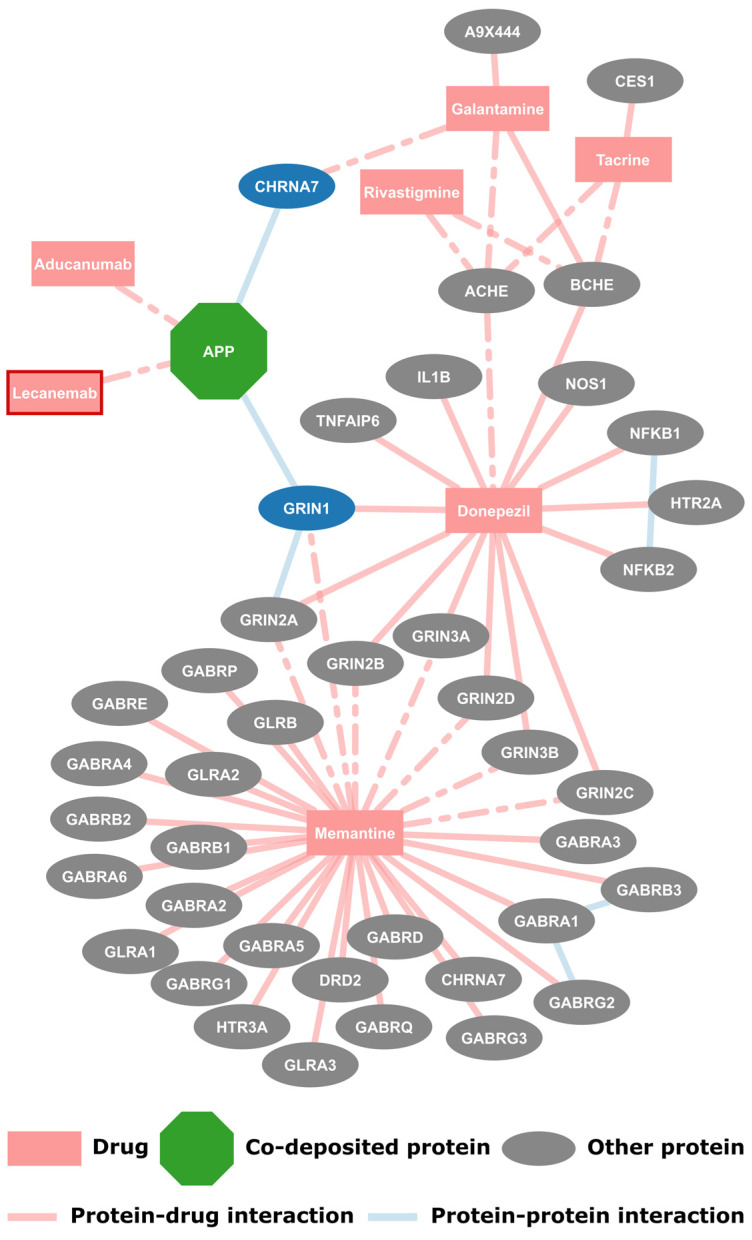
**Network of the drugs approved by the FDA for use in AD and their protein targets**. Of the 12 proteins found on amyloid plaques, only APP is a protein target of some of these drugs. Additionally, there are no first neighbors of co-deposited proteins aside from the two proteins APP interacts with shown here in blue. Drug–protein interactions where DrugBank reports a pharmacological action are shown with a dashed line, while a solid line is used for those categorized as unknown. The latest approved drug lecanemab is shown with a red border.

The first step for this work was to create a drug-protein interaction network for the drugs approved by the FDA for use in AD. Originally 6 drugs were collected, namely donepezil, rivastigmine, galantamine, memantine and aducanumab, along with one withdrawn drug, tacrine. As of the writing of this paper another drug, lecanemab, was approved and was manually added to this dataset, bringing the total of drugs for AD to seven (Figure 1, shown with a red border). The protein targets of the AD drugs were extracted from DrugBank along with the information of whether there is known pharmacological action. Additionally, the interactions between these target proteins were collected from IntAct. The resulting network consists of 49 nodes and 62 edges that form single component, as shown in Figure 1. Each drug, with the exception of the two monoclonal antibodies, has multiple protein targets, however they have known pharmacological action only on a subset of those proteins. The targeted proteins are mostly related to neurotransmission, such as G-protein-coupled receptors or cholinesterases. It is noted that APP is the only protein found on amyloid plaques in this network, as it is targeted by the aforementioned antibodies. Here, APP interacts with two other proteins, targets of *galantamine* and *donepezil*, respectively; these proteins are also the only first neighbors of co-deposited proteins found here.

### 2.2. Proteins Found on Amyloid Plaques

**Figure 2:**
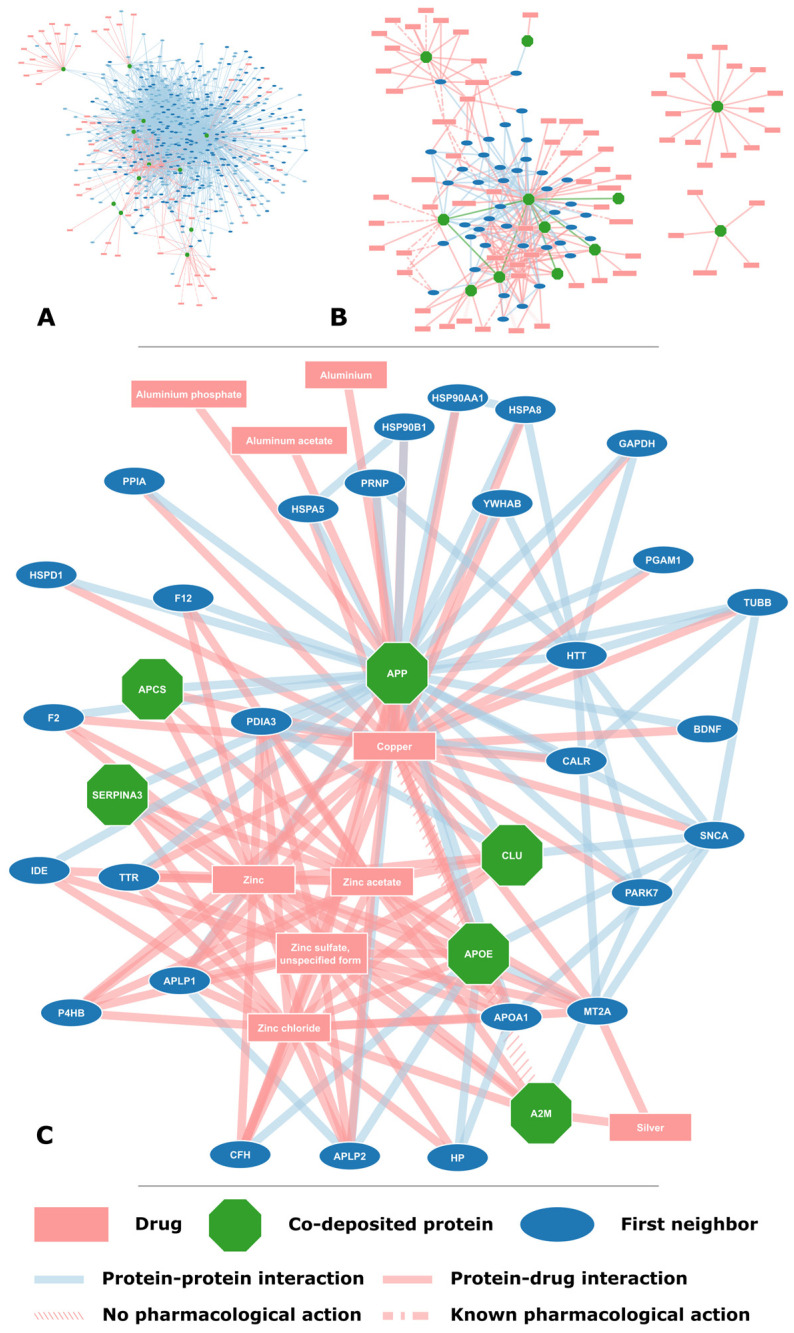
**Protein–protein and protein–drug interaction networks for the proteins found on amyloid plaques.** (**A**) The network consisting of all first neighbors and drugs targeting the co-deposited protein. (**B**) Some of the drugs targeting these proteins also target a number of other proteins, some of which are first neighbors. Isolated to the right are the two cathepsins D and B, interacting almost exclusively with substances used for the determination of their 3D structure (not actual drugs). (**C**) Essential elements and related substances, specifically zinc and copper, interact with a multitude of proteins in the network. Here, as in the other networks, APP is shown to have a central role interacting with the majority of proteins in the network.

The focus of this study is the proteins found on amyloid plaques, the key pathological characteristic in AD; a total of 12 proteins found in amyloid plaques were extracted from AmyCo (Table 1). Their protein interactions were collected from IntAct and all proteins had a minimum of two first neighbors. In total, 525 proteins were collected, including the AmyCo proteins, that had 1994 interactions between them (Supplementary File S1). Few interactions appear to take place between the co-deposited proteins with only 5 proteins interacting with APP and one interaction between tau and apolipoprotein E. However, with the inclusion of first neighbors and drugs the resulting network does form a single connected component (Figure 2A).

According to the literature, most of the proteins found on amyloid plaques have a well-established role in AD, but specific underlying mechanisms are still under investigation. APP and MAPT are the precursor proteins whose amyloids make up the amyloid plaques and neurofibrillary tangles, respectively. APOE, an apolipoprotein key in cholesterol transport, is the strongest genetic risk factor for sporadic AD and has a key role in amyloid-β metabolism and clearance [10]. A2M is a protease inhibitor abundant in serum that has been implicated in amyloid-β metabolism and genetically linked to AD [21]. Similarly, another protease inhibitor, SERPINA3, has been associated with AD with evidence suggesting it promotes AD development [22]. Impaired function of proteases CTSD and CTSB has been associated with amyloid-β accumulation and AD [23,24]. CLU is an extracellular chaperone associated with AD both as a genetic factor and as a key factor in amyloid-β aggregation [8]. IL6 is a mediator of inflammation whose signaling has been associated with cognitive impairment in AD [25]. APCS, also known as SAP, is a serum protein that is found in amyloid deposits, such as amyloid plaques, and is a promoter of amyloid-β aggregation and AD pathogenesis [26]. HSPG2 is part of the extracellular matrix that accelerates amyloid-β fibril formation and is linked to AD [27]. CST3 is a protease inhibitor that has a neuroprotective role in AD by inhibiting amyloid-β aggregation [28].

The drugs targeting the 12 proteins (Table 1) were extracted from DrugBank and totaled 72 drugs; only cystatin-C was not targeted by any drug (Supplementary File S2). Some of the substances included in DrugBank are PDB [29] ligands or modified residues used for the experimental determination of 3D protein structure, such as most drugs reported as targeting cathepsins D and B. The protein–drug interactions were incorporated in the protein–protein interaction network, resulting in a network of 525 proteins, 72 drugs and 2191 edges. Some of the drugs targeting co-deposited proteins had multiple targets, including some first neighbors (Figure 2B). In particular, drugs that are forms of essential elements, such as zinc and copper, tended to have numerous targets (Figure 2C).

### 2.3. Potential for Drug Repurposing

**Figure 3:**
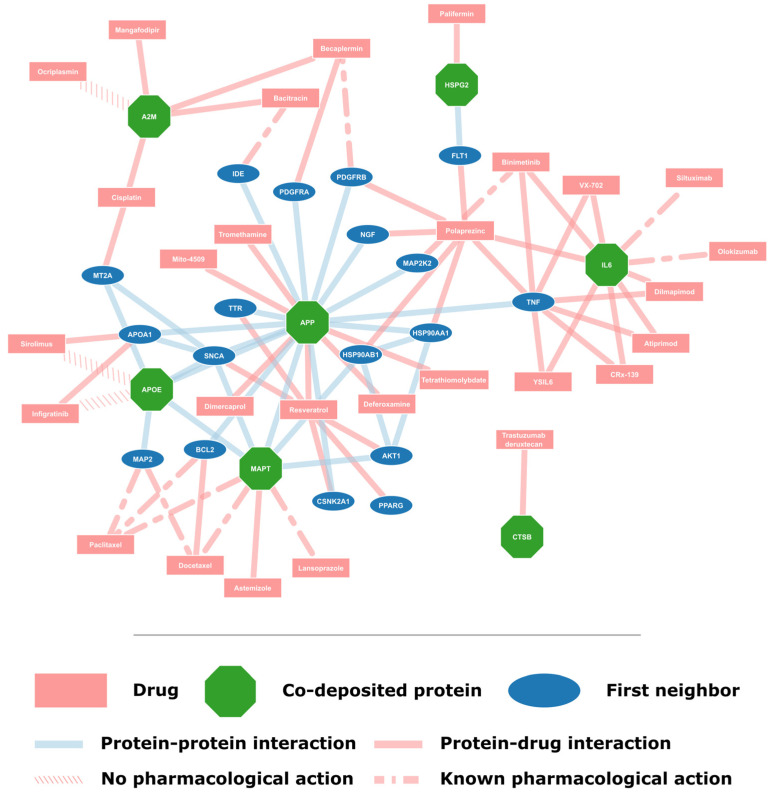
**Drug candidates for repurposing and their protein targets.** In total, 28 drugs are proposed as potential candidates for use in AD (Table 2). Protein targets in this network are the proteins found in amyloid plaques and any of their first neighbors that are targeted by these drugs. Protein–drug interactions are shown according to the pharmacological actions according to DrugBank.

Information was extracted for each drug targeting the co-deposited proteins from DrugBank and the literature. The type of each drug was determined, e.g., monoclonal antibody, and relevant references from DrugBank were extracted. Moreover, to explore the potential for repurposing these drugs for use in AD, a search on PubMed was done to find known correlations between the drug and AD. The collected information is available in the Supplementary File S2. Potential candidates for repurposing are shown in Table 2; drugs used for AD, those related to essential elements and substances used to determine the 3D structure of proteins were excluded. The network of protein–drug interactions for these candidate drugs is shown in Figure 3. Lastly, the drugs and proteins collected in this study were compared with the proposed drugs or drug targets suggested by similar previous studies [15,16,17,18,19,20] (Supplementary File S2).

## 3. Discussion

The search for an efficient AD treatment has been ongoing for many years with little to no results. As of the start of this study only five drugs had been approved by the FDA for use in AD [30]. These were the acetylcholinesterase inhibitors, *donepezil*, *rivastigmine*, *galantamine* and the now-withdrawn *tacrine*, and an antagonist of the N-methyl-D-aspartic acid (NMDA) receptor, *memantine*. These drugs do not halt or slow the progression of AD but are rather prescribed with the aim of providing palliative care. For almost two decades after the approval of *memantine* no other drug would be approved for use in AD. Then, in June 2021, and in what is considered by many as a controversial decision, the FDA approved the drug *aducanumab*, a monoclonal antibody targeting amyloid-β aggregates [31]. Now, during the writing of this publication, the FDA is approving yet another drug, *lecanemab*, with a similar mechanism of action to *aducanumab*.

Despite the recent approvals of the two monoclonal antibodies targeting amyloid-β aggregates, questions regarding potential risks and benefits remain. However, it is obvious that amyloid-β remains a favorite candidate target for drugs against AD, even as the AD drug pipeline is diversifying [32]. The importance of amyloid-β has been supported for a long time as it is the main component of amyloid plaques, a pathological hallmark of AD. More recently, it has been shown that amyloid plaques contain other components apart from amyloid-β; such proteins are called co-deposited and their role is as of yet unclear. We suggest that these proteins are likely promising pharmaceutical targets for AD treatment.

To explore this idea, a network of protein–protein and protein–drug interactions was investigated. The proteins found on the amyloid plaques present in AD were collected from AmyCo, a manually curated database of amyloidosis and other conditions related to the deposition of amyloids. In total, 12 proteins were collected, including the key proteins for the formation of amyloid deposits, namely APP and tau. It is noteworthy that more than half of the co-deposited proteins do not have reported interactions in IntAct with APP, or its produced peptides. Possibly, the interaction is not direct, but rather mediated through other proteins, which can be supported by the formation of a single connected component in the protein network. Alternatively, some proteins may interact specifically with the aggregated or “diseased” form of amyloid-β while protein interaction databases primarily consist of interactions between soluble or “normal” proteins. Either way, it would be informative to experimentally investigate if co-deposited proteins interact directly with the key component or the precursor protein, in this case amyloid-β and APP, as well as to see if any of their protein neighbors are found on amyloid plaques. However, this is a limitation of this work as only a limited subset of the protein–protein interactions in humans is available in interaction databases.

Beyond their presence on amyloid plaques, co-deposited proteins are showcasing their importance based on a variety of drugs that target them. None but one of the drugs approved for AD by the FDA were amongst these drugs. This was expected as the early drugs for AD were oriented towards the alleviation of symptoms rather than addressing the cause of AD. Few of the drugs targeting co-deposited proteins are used for AD, such as the anti-amyloid antibody *aducanumab* or imaging agents. Some were substances used for determining the 3D structure of proteins. Several drugs which tended to have multiple targets in the network were related to essential elements, such as copper and zinc; these have been associated with AD and are considered as potential therapeutic targets [33]. Lastly, there is a variety of drugs used for other diseases.

In this latter category of drugs that target co-deposited proteins exist potential targets for drug repurposing for use in AD. In fact, some of these drugs have already been associated with AD. Resveratrol, an antioxidant and anti-inflammatory substance that has displayed neuroprotective properties, is considered for the treatment of AD [34] and has been tested via clinical trial [35]. Anti-copper agent tetrathiomolybdate has been proposed as a potential agent for addressing the copper imbalance in AD [36] and a study on mouse model showed tetrathiomolybdate’s promise for use in AD prevention [37]. Similarly, a study on mice tested the use of microtubule stabilizing drugs and showed potential for such drugs [38], like the anti-cancer drugs paclitaxel and docetaxel [39]. Another interesting drug is lansoprazole, a proton pump inhibitor used for gastrointestinal issues, that has been suggested as a neuroimaging agent due to its selective interaction with tau polymers [40] but has also been linked to enhanced amyloid-β production [41]. Lastly, this work can be expanded to the numerous drugs targeting first neighbors that can be investigated for possible use in AD (Supplementary File S1). To this end, a simple web application with interactive networks was created (http://bioinformatics.biol.uoa.gr/AD_drugs/, accessed on 19 April 2025).

The results of this study were compared to previous studies with a similar methodology of using networks for drug discovery. Siavelis et al. suggested 27 drugs structurally distinct to those that were currently used [18]. Han et al. tried to find genes that can be used as drug targets for AD; they narrowed down their data to 18 target genes [17]; these include two co-deposited proteins: A2M and HSPG2 (Table 1). Lee et al. used drug induced gene perturbation signatures to arrive at 32 drugs [20]; of these, two were also found in this study: paclitaxel and deferoxamine. Peng et al. determined which drugs are close to AD and narrowed them down to 24 drugs after testing them on neural cell lines [19]. Soleimani Zakeri et al. suggested 14 drugs that were associated with AD genes [15]. Savva et al. used structural similarity to arrive at 26 elite drugs [16]. Overall, little to no overlap was found between the results of our study and previous studies (Supplementary File S2). This supports the novelty of our strategy to focus on the co-deposited proteins.

Following this work, an evaluation of the proposed drug candidates will be necessary. On a bioinformatics front, information can be collected about these drugs regarding their experimental or predicted properties (e.g., blood–brain-barrier permeability) through DrugBank [42] and other sources. Next, on a structural level, molecular docking and molecular dynamics studies can be used to assess drug-target affinity (e.g., [43,44]). Other network analyses can be done to determine appropriate model organisms based on the target protein and pathways (e.g., [45]). Once the most promising candidates are identified, experimental studies on model organisms and cell assays will be required.

## 4. Materials and Methods

### 4.1. Co-Deposited Proteins

The proteins found to be co-deposited in amyloid plaques were collected from the AmyCo database [7]. AmyCo is a collection of amyloidosis and other clinical disorders related to the deposition of amyloids. Each disease entry consists of some basic information, including the precursor amyloid protein and a number of co-deposited proteins, and cross-references. A protein is entered as co-deposited if there is experimental evidence verifying their presence in amyloid plaques via MS-based proteomics, immunohistochemistry, staining or imaging techniques [7]. Specifically, for this study 12 proteins were extracted from the disease entry corresponding to “Alzheimer disease”, the precursor (amyloid-β precursor protein-APP) and another 11 co-deposited proteins.

### 4.2. Drugs and Their Protein Targets

Firstly, the drugs approved by the FDA for use in AD were found and their protein targets were collected from DrugBank 5.0 [42]. DrugBank is a comprehensive database with information on drugs and their targets, including the pharmacological action on each target if known. This network of FDA approved drugs and targets is to be contrasted against the network of proteins found in amyloid plaques. Drugs that target these 12 proteins were extracted from the UniProt (version 2022_02) [46] text files (version 2022_02), from the cross-reference database DrugBank 5.0. Additionally, DrugBank was queried directly to validate the list of drugs targeting co-deposited proteins collected from UniProt and to gather further information on drugs and their protein targets. Finally, a review of the literature was undertaken to collect information about any known association between each drug and AD or related processes.

### 4.3. Protein-Protein Interactions

Protein-protein interactions were retrieved from IntAct (accessed on 1 June 2022) [47], a database of curated experimentally validated molecular interactions. To collect the first neighbors/interactors of the co-deposited proteins, their UniProt Accession Numbers were used to search IntAct. All interactions between co-deposited and/or first neighbors were gathered for a complete network. The results were filtered to exclude non-human and non-protein interactors, as well as interactions with low confidence score (<0.45) [48]. Lastly, all self-loops were removed from the dataset.

### 4.4. Network Visualization

The popular tool Cytoscape (version 3.10.3) [49] was used for integrated molecular network visualization and analysis. An advantage of using Cytoscape is the array of Apps available, including the IntAct App [50] which allows the import of networks directly from the IntAct database. Lastly, Cytoscape.js, a JavaScript library, was used to create a webpage (http://bioinformatics.biol.uoa.gr/AD_drugs/ (accessed on 23 February 2025)) to allow the easy exploration of networks by other researchers.

## 5. Conclusions

New drugs for AD are getting approved by the FDA for the first time in almost two decades, but the search for safe and effective treatment goes on. Amyloid plaques are the pathological hallmark of AD, and the co-deposited proteins found there have thus far been mostly unexplored. In this work, we used protein–protein and protein–drug interaction networks of these proteins to showcase their potential for drug repurposing. As a result of this pipeline analysis, a list of 28 drugs with a potential for use in AD is reported, half of which have already been associated with AD and related processes. Further computational and experimental studies are required to validate their potential usage as AD therapeutics.

## Figures and Tables

**Table 1:** **The proteins found on amyloid plaques according to the AmyCo database.** The first three columns are the accession number, gene name and protein name according to UniProt. Following are the numbers of first neighbors (proteins) according to IntAct and drugs that target that protein from DrugBank. The last column indicates whether the protein directly interacts with APP.

Gene Name	Accession	Protein Name	Neighbors	Drugs	Interacts with APP
**APP**	P05067	Amyloid-β precursor protein	386	22	-
**A2M**	P01023	Alpha-2-macroglobulin	55	11	no
**APOE**	P02649	Apolipoprotein E	43	7	yes
**MAPT**	P10636	Microtubule-associated protein tau	39	5	yes
**CTSD**	P07339	Cathepsin D	22	5 *	no
**CLU**	P10909	Clusterin	10	5	yes
**SERPINA3**	P01011	Alpha-1-antichymotrypsin	8	4	yes
**CTSB**	P07858	Cathepsin B	5	15 *	no
**IL6**	P05231	Interleukin-6	5	13	no
**APCS**	P02743	Serum amyloid P-component	3	6	yes
**HSPG2**	P98160	Basement membrane-specific heparan sulfate proteoglycan core protein	3	1	no
**CST3**	P01034	Cystatin-C	2	-	no

* Most of these are PDB ligands.

**Table 2:** **Drug candidates for repurposing to be used in the treatment of AD.** Drugs in the list to the left (15) have a known association with AD or related processes, while little to no such information is available for those on the right (13).

Associated with AD and Related Processes			No or Limited Known Association with AD		
DrugBank ID	Drug Name	Type	DrugBank ID	Drug Name	Type
DB00626	Bacitracin	antibiotic	DB05470	VX-702	anti-cytokine
DB00637	Astemizole	antihistamine	DB05744	CRx-139	anti-inflammatory
DB02709	Resveratrol	antioxidant	DB12140	Dilmapimod	anti-inflammatory
DB06782	Dimercaprol	chelator	DB09221	Polaprezinc	antioxidant
DB05088	Tetrathiomolybdate	chelator	DB11886	Infigratinib	cancer treatment
DB00515	Cisplatin	chemotherapy	DB00746	Deferoxamine	chelator
DB01229	Paclitaxel	chemotherapy	DB05513	Atiprimod	chemotherapy
DB01248	Docetaxel	chemotherapy	DB11967	Binimetinib	chemotherapy
DB05846	Mito-4509	estrogen	DB06796	Mangafodipir	contrast agent
DB00877	Sirolimus	immunosuppressant	DB13127	Olokizumab	monoclonal antibody
DB00102	Becaplermin	protein based therapy	DB09036	Siltuximab	monoclonal antibody
DB08888	Ocriplasmin	protein based therapy	DB14962	Trastuzumab deruxtecan	monoclonal antibody
DB00039	Palifermin	protein based therapy	DB05017	YSIL6	T-cell inhibitor
DB03754	Tromethamine	proton acceptor			
DB00448	Lansoprazole	proton pump inhibitor			

## Data Availability

All necessary data are available in the Supplementary Material.

## Supplementary Materials

The following supporting information can be downloaded at: https://www.mdpi.com/article/10.3390/molecules30081736/s1, Supplementary File S1: Protein–protein and protein–drug interactions; Supplementary File S2: Potential candidates for drug repurposing.

## Abbreviations

The following abbreviations are used in this manuscript:

AD	Alzheimer’s Disease
APOE	Apolipoprotein E
